# Population migration, spread of COVID-19, and epidemic prevention and control: empirical evidence from China

**DOI:** 10.1186/s12889-021-10605-2

**Published:** 2021-03-17

**Authors:** Zhen Hu, Yuanyang Wu, Mohan Su, Lin Xie, Anqi Zhang, Xueyu Lin, Yafeng Nie

**Affiliations:** 1 School of Economics and Management, Northwest Agricultural and Forestry University, Xianyang, China; 2 School of Public Administration, Zhongnan University of Economics and Law, Wuhan, China; 3 Institution of Population and Labor Economics, The Chinese Academy of Social Science, Beijing, China; 4 School of Public Economy and Management, Shanghai University of Finance and Economics, Shanghai, China

**Keywords:** Wuhan, COVID-19, Population migration, Epidemic prevention and control

## Abstract

**Background:**

This study applied the susceptible-exposed-infectious-removed (SEIR) model to analyze and simulate the transmission mechanisms of the coronavirus disease 2019 (COVID-19) in China.

**Methods:**

The population migration was embedded in the SEIR model to simulate and analyze the effects of the amount of population inflow on the number of confirmed cases. Based on numerical simulations, this study used statistical data for the empirical validation of its theoretical deductions and discussed how to improve the effectiveness of epidemic prevention and control considering population migration variables. Statistics regarding the numbers of infected people in various provinces were obtained from the epidemic-related data reported by China’s National Health Commission.

**Results:**

This study explored how the epidemic should be prevented and controlled from the perspective of population migration variables. It found that the combination of a susceptible population, an infected population, and transmission media were important routes affecting the number of infections and that the migration of a Hubei-related infected population played a key role in promoting epidemic spread. Epidemic prevention and control should focus on regions with better economic conditions than the epidemic region. Prevention and control efforts should focus on the more populated neighboring provinces having convenient transportation links with the epidemic region. To prevent and control epidemic spread, priority should be given to elucidating the destinations and directions of population migration from the domestic origin of infections, and then controlling population migration or human-to-human contact after such migration.

**Conclusions:**

This study enriched and expanded on simulations of the effects of population migration on the COVID-19 epidemic and China-based empirical studies while offering an epidemic evaluation and warning mechanism to prevent and control similar public health emergencies in the future.

## Background

In December 2019, it was reported that patients with pneumonia of unknown etiology had been in the Huanan Seafood Wholesale Market in Wuhan, Hubei province. These patients, who were admitted to the Wuhan Jinyintan Hospital for treatment and were later diagnosed with coronavirus disease 2019 (COVID-19). Following this incident, COVID-19 has gained prominence, globally [1–4]. With the large-scale human migration during the Chinese New Year, COVID-19 spread rapidly in China. As of May 2, 2020, there had been 84,391 confirmed cases and 4643 deaths in China, with a mortality rate of 5.5%. Among them, 50,332 cases were reported in Wuhan, where the mortality rate was as high as 7.69%. Based on epidemiological theories, the combination of a susceptible population, an infected population, and transmission media are important channels affecting the number of infections. In particular, the migration of exposed and symptomatic infected people during the incubation period play a key role in promoting the spread of the disease. In order to control population migration, various Chinese provinces and cities successively activated Level-1 Response to Major Public Health Emergencies, carried out joint prevention and control measures, instituted work and production stoppages, and also enacted stringent lockdowns rules in all urban and rural communities [5–7]. These measures eventually succeeded in controlling China’s epidemic.

While China was seeing the first evidence of success in its epidemic control efforts, increasingly, the epidemic spread to countries, worldwide, due to international population migration in the context of globalization. As of May 2, 2020, 192 countries and regions globally had reported COVID-19 cases, with the cumulative confirmed cases totaling 3.33 million and a death toll exceeding 245,400. The mortality rate was about 7.3% globally 14% in the UK and France. Although countries directed efforts against the COVID-19 epidemic, the outcome remains poor [8]. In the face of this global public health crisis, Bruce Aylward, senior advisor to the Director-General of the World Health Organization (WHO), called for countries to strengthen their collaboration and management in aspects such as scientific research, population movement, and the supply of medical resources and to add greater synergy to the global battle against the challenges posed by COVID-19. In view of the initial success achieved by China in COVID-19 prevention and control, Tedros Adhanom, the WHO’s Director-General, urged countries around the world to learn from China’s experience [9]. In view of China’s anti-epidemic measures, the organization, and mobilization capabilities of the government, which manifested especially in the lockdowns and other prevention and control measures against population migration; have attracted widespread attention of other countries [3, 10–13]. To communicate China’s experience in the global fight against COVID-19 and to respond to similar public health emergencies in the future, there is a need for China to summarize the patterns of the disease progression and their anti-epidemic experience of this event [14, 15], with an emphasis on the mechanism through which population migration affected the epidemic spread.

Contacts between infected and susceptible populations are important channels for disease transmission, and migration plays a key role in promoting their contacts. In this study, we used population migration perspective to answer two questions: 1) How did population migration affect the number of confirmed cases in each region and what were the epidemic transmission patterns? 2) Based on theoretical and empirical analyses of the effects of population migration on the number of confirmed cases, what were the factors influencing population migration, and how should a warning system be constructed for the prevention and control of the current epidemic and any similar future event? The specific contents of this study are as follows: First, this study applied the susceptible-exposed-infectious-removed (SEIR) model to simulate the effects of the amount of Hubei-related population inflow on the number of infections in various Chinese provinces, followed by an analysis of the factors influencing population migration. Second, in this study, we utilized population migration data and statistics of confirmed cases to empirically prove the theoretical deductions in the previous section. Third, this study elaborated on how to construct an epidemic evaluation and warning system based on population migration variables.

This study is distinctive from the studies by Wu et al. [16], Zhao and Chen [17], Tang et al. [18], Anastassopoulou et al. [19], Yang and Wang [20], Chen et al. [21] and Huang et al. [22] in the following ways: First, population migration was embedded in the SEIR model to simulate and analyze the effects of the amount of population inflow on the number of confirmed cases. Second, this study, compared with others which are based on numerical simulations, used statistical data for the empirical validation of its theoretical deductions. Finally, in addition to the existing numerical simulations and empirical studies performed by others, this study also discussed how to improve the effectiveness of epidemic prevention and control based on population migration variables. The contribution of this study is its potential to enrich and expand knowledge on the simulations of the effects of population migration on the COVID-19 epidemic and China-based empirical studies. At the same time, it provided an epidemic evaluation and warning mechanism based on population migration variables to prevent and control similar public health emergencies in the future. In addition, it summarized China’s epidemic prevention and control measures based on population migration variables to assist in the global disease prevention and control efforts that are underway.

## Methods

### Theoretical analysis

#### Construction of SEIR model

The studies by Chen et al. [21], Yang and Wang [20], Anastassopoulou et al. [19], Tang et al. [18], Zhao and Chen [17], Wu et al. [16], Huang et al. [22], and Wan et al. [23] were used as references for the SEIR model settings in the present study. The model settings are as follows. Considering that COVID-19 has an incubation period and assuming that populations were divided into five compartments, *S*_*t*_*, E*_*t*_*, I*_*t*_, *R*_*t*_, and *D*_*t*_ which denote the sizes of the susceptible populations, the population in the incubation state, the infected population, the recovered population, and the deceased population at time *t* respectively. The total number of people in the system, *N*, was a constant and assumed to be *N* ≡ *S*_*t*_ + *R*_*t*_ + *E*_*t*_ + *I*_*t*_ + *D*_*t*_.

Assuming that individuals in the incubation and infected states came into contact with *m*_1_ and *m*_2_ number of people daily, on average, that the levels of transmissibility in these two groups were *β*_1_ and *β*_2_ respectively, and that the daily numbers of people infected by individuals in the incubation and infected states were *m*_1_*β*_1_*ES*/*N* and *m*_2_*β*_2_*IS*/*N* respectively, then the rate of change in the size of the susceptible population was defined as follows:

1 $$ \frac{dS}{dt}=-{m}_1{\beta}_1 ES/N-{m}_2{\beta}_2 IS/N $$

Assuming that individuals in the incubation state converted to infected individuals daily at a probability of *α*, then the rate of change in the size of the population in the incubation state was defined as follows:

2 $$ \frac{dE}{dt}={m}_1{\beta}_1 ES/N+{m}_2{\beta}_2 IS/N-\alpha E $$

Assuming that infected individuals converted to the recovered and deceased states daily at a probability of *γ*_1_ and *γ*_2_ respectively, then the rates of changes in the sizes of the infected population, recovered population, and deceased population were defined as follows:

3 $$ \frac{dI}{dt}=\alpha E-{\gamma}_1I-{\gamma}_2I $$

4 $$ \frac{dR}{dt}={\gamma}_1I $$

5 $$ \frac{dD}{dt}={\gamma}_2I $$

In summary, the SEIR model was defined as follows:

6 $$ \left\{\begin{array}{c}\frac{dS}{dt}=-{m}_1{\beta}_1 ES/N-{m}_2{\beta}_2 IS/N\\ {}\frac{dE}{dt}={m}_1{\beta}_1 ES/N+{m}_2{\beta}_2 IS/N-\alpha E\\ {}\frac{dI}{dt}=\alpha E-{\gamma}_1I-{\gamma}_2I\\ {}\frac{dR}{dt}={\gamma}_1I\\ {}\frac{dD}{dt}={\gamma}_2I\end{array}\right. $$

According to Geng et al. [24], *β*_1_ = *β*_2_ = 0.045. According to Fan et al. [25], *α* = 1/7. Based on the reported COVID-19 data by China’s National Health Commission, data were publicly available, the proportion of recovered patients and the mortality rate fluctuated at 0.049–0.085 and 0.0009–0.0015 respectively between 1 and 6 March 2020, and the mean values of the intervals were obtained so that *γ*_1_ = 0.069 and *γ*_2_ = 0.00115. Assuming that *I*_*t*_, *R*_*t*_, and *D*_*t*_ were all 0 initially, *N* = *E*_0_ + *S*_0_ = 1. Considering the complexity of the solution process, the results of the numerical simulation are shown directly.

#### Theoretical analysis of the effects of population migration

**Fig. 1:**
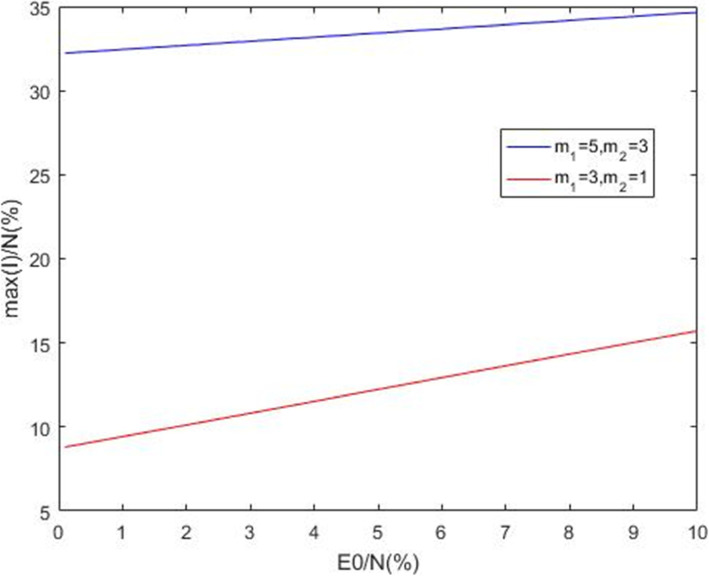
Effects of population migration from the epidemic region on the number of infected people

After the onset of the outbreak, two groups of people migrated from Hubei to other provinces. These were permanent Hubei residents from other provinces who returned home during the Chinese New Year and permanent local residents of Hubei Province who travelled to visit relatives or as outbound tourists during the Chinese New Year. Both groups migrated to other provinces during the COVID-19 outbreak. The number of potential infections during such migration mainly affected the initial number of people in the incubation state in each city, *E*_0_. Hence, an analysis of the effects of the Hubei-related population migration on the number of infections in other provinces resolved itself into an analysis of the effects of the initial number of people in the incubation state, *E*_0_, on the number of infected people, *I*(*t*). Considering that the number of infected people was dynamic and that the trajectory of the number of newly infected people displayed an inverted *U* shape, only the effects of the initial number of people in the incubation state on the peak number of infected people were obtained. To ensure the robustness of the conclusion, simulation studies were carried out under two states: frequent and infrequent public interaction. When public interaction was frequent, *m*_1_ = 5 and *m*_2_ = 3. When public interaction was infrequent, *m*_1_ = 3 and *m*_2_ = 1. The simulation results are shown in Fig. 1, where the y-axis is the peak number of infected people, and the x-axis is the proportion of Hubei-related population inflow in the total population of a given province.

As shown in Fig. 1, under both frequent and infrequent public interactions, a greater number of people migrating from Hubei to other provinces, i.e., a greater initial number of people in the incubation state, was associated with a higher peak number of infected people. The peak number of infected people was higher when there was frequent public interaction, compared to when there was less frequent interaction. In conclusion, the theoretical analysis revealed that the greater the initial number of people who migrated from Hubei after the outbreak, the greater the number of infected people in other provinces at a later stage. To validate the aforementioned proposition from the empirical angle, the next section analyzed the empirical relationship between the number of people who migrated from Hubei to other provinces, or the number of those who migrated from other provinces to Hubei, and the number of infected people in those provinces.

The above theoretical analysis revealed that contact between the infected and susceptible populations was the main cause of epidemic spread. In the context of China’s large-scale population migration during the Chinese New Year, migration of infected people from Hubei played a key role in determining the speed of epidemic spread. The answers to questions such as whether these theoretical deductions were empirically valid, how population migration influenced the number of infected people in various Chinese provinces, and which variables were influencing population migration would help to prevent and control the current epidemic and establish warning indicators for similar epidemics in the future. In the next section, empirical validation was conducted to substantiate the theoretical deductions made above.

### Empirical validation

#### Model settings

Based on the theoretical analysis, the number of infected people was mainly affected by the combined effects of factors including the number of susceptible people, the number of infected people, transmission media, medical conditions, and immunization measures. The number of infected people in another province (*Y*) was regarded as the dependent variable. The number of susceptible people was equated roughly to the number of permanent residents in each province. Considering that the initial number of infected people was associated with Hubei, the size of a Hubei-related population could be equated roughly to the number of people infected with the virus. Thus, the number of permanent residents in each province (*P*) were taken as the number of susceptible people. Considering that the outbreak site of COVID-19 was Hubei Province, the number of people who migrated from Hubei to each province (*PHM*)and the number of people who migrated from each province to Hubei (*PMH*) were taken as the major source of infection in each province. Population density (*PD*) and traffic density (*TD*) were considered to be the transmission media for the epidemic. Also, the proportion of population over 65 years old (*Over*65),and whether to start the first level response before January 24 (*Emergency*) were taken as the control variables in the model. This generated the susceptible population, the infected population, and transmission media in the theoretical model mentioned above.

In addition, this study also shed light on factors affecting population migration from Hubei to other provinces and vice versa. By drawing on the studies by Karemera et al. [26], Piore [27], Stark and Taylor [28], Cai and Wang [29], Shen [30], Henry et al. [31], Fan [32], Zhu and Chen [33], and Borrow [34] on the factors influencing population migration, the following variables were selected as the control variables: the number of permanent residents in each province, whether the province neighbors Hubei (*neighbor*), whether the province is in Southern China (*region*), the number of high-speed trains between the province and Hubei (*QHrail*), the geographical distance between the province and Hubei (*distan*), the comparison of disposable income per capita between the province and Hubei (*comparison*), and the comparison of the number of 5A-level scenic spots between the province and Hubei (*FiveA*).The definition of each variable is shown in Table 1. The number of infected people and the model of population migration were defined as follows:

9 $$ {Y}_i=\alpha +{\beta}_i{X}_i+{\mu}_i{Z}_i $$

10 $$ {M}_i=c+{\mu}_i{D}_i+{\theta}_i $$

where *i* represents the *i* ^th^ province; *Y*_*i*_ is the main explanatory variable; *M*_*i*_ = (*PHM*_*i*_, *PMH*_*i*_, *PTH*_*i*_) is the explained variable of the model (10); *X*_*i*_ = (*P*_*i*_, *PHM*_*i*_, *PMH*_*i*_) is the main explanatory variable of the model (9); *Z*_*i*_ = (*PD*_*i*_, *TD*_*i*_, *Over*65, *Emergency*_*i*_) is the control variable of the model (9); and *D*_*i*_ = (*P*_*i*_, *neighbor*_*i*_, *region*_*i*_, *QHrail*_*i*_, *dis*tan_*i*_, *FiveA*_*i*_) denotes the explanatory variable of model (10). *β*_*i*_, *β*_*i*_, and *μ*_*i*_ represent the parameters to be estimated for models (9) and (10). *ε*_*i*_ and *θ*_*i*_ denote the random error terms, namely, other factors affecting the number of infected people.

**Table 1:** Descriptive statistics of variables

Variable	Definition	Obs	Mean	Std.	Min	Max
NCOV	Number of infected people (people)	31	2619.581	12,164.41	1	68,127
PHM	Number of people who migrated from Hubei to another province (10,000 people)	30	31.87	74.059	0.85	397.34
PMH	Number of people who migrated from another province to Hubei(10,000 people)	30	9.891	9.973	0.6	44.89
P	Number of permanent residents in each province (10,000 people)	31	4395.032	2797.833	318	10,724
PD	Population density, number of people per km^2^ (people)	31	453.376	705.27	2.59	3825.99
TD	Traffic density, mileage per km^2^ (km)	31	0.955	0.571	0.06	2.46
neighbor	Whether it neighbors Hubei, yes = 1, no = 0	30	0.233	0.43	0	1
region	Whether it is in southern China, yes = 1, no = 0	30	0.467	0.507	0	1
QHrail	Number of high-speed trains between the provincial capital and Wuhan (trains)	30	21.1	26.61	0	99
distance	Distance from Hubei (km)	30	1230.333	721.342	327.1	3263.8
comparison	Whether the disposable income per capita is higher than that in Hubei, yes = 1, no = 0	31	0.387	0.495	0	1
Over65	the proportion of population over 65 years old	31	0.096	0.02	0.05	0.14
FiveA	the comparison of number of 5A-level scenic spots between the province and Hubei	31	0.226	0.425	0	1
Emergency	whether to start the first level response before January 24	31	0.581	0.502	0	1

**Notes:** Data were obtained from the National Health Commission of China (as of May 2, 2020), 2015 China 1% National Population Sample Survey, 2015 China Statistical Yearbook, Ministry of Land and Resources of China, official website of the China Railway Corporation, and local government website. The research area of this article covers 31 provinces, municipalities, and autonomous regions in China

#### Data sources

**Fig. 2:**
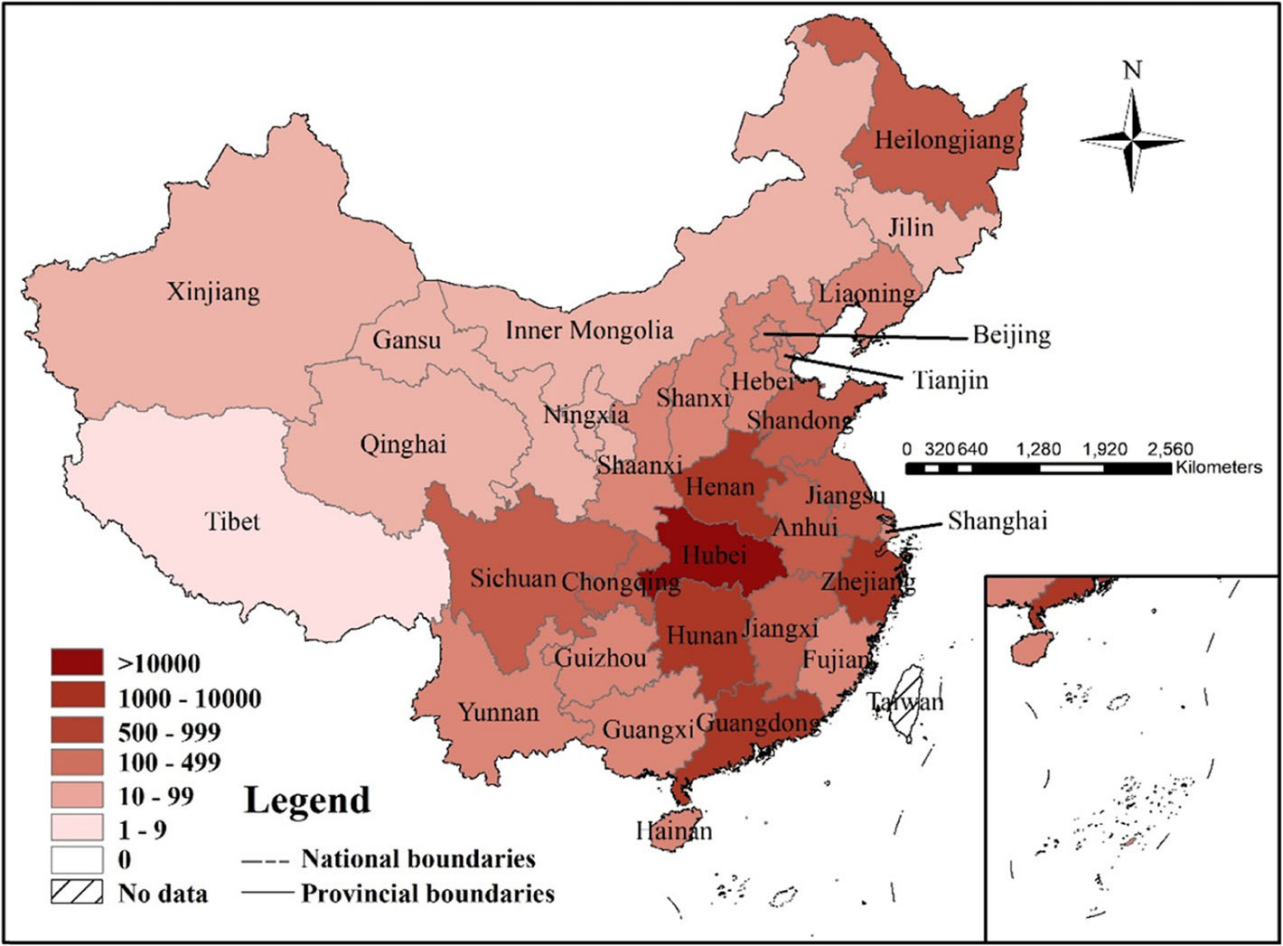
The number of infected people according to Chinese Province (as of 2 May 2020)

Statistics regarding the numbers of infected people in various provinces were obtained from the epidemic-related data reported by China’s National Health Commission. The number of people who migrated from Hubei to other provinces and vice versa was obtained from the 2015 China 1% National Population Sample Survey [35]. The number of permanent residents, transport mileage, and disposable income per capita for each province, the proportion of population over 65 years, and the number of 5A-level scenic spots were sourced from the 2015 China Statistical Yearbook [36]. The surface areas of various provinces were obtained from the Ministry of Land and Resources of China [37]. The Qinling–Huaihe Line was used as a reference line to distinguish between Northern and Southern China, with provinces to the north of this line as northern provinces and those to the south as southern provinces. In terms of the number of inter-provincial high-speed trains, only the number of high-speed trains operating between each provincial capital and Wuhan was considered. The official website of the China Railway Corporation (12,036.cn) was used to obtain the number of high-speed trains between each province and Wuhan. As for the other variables, the population density was obtained by dividing the number of permanent residents in each province by the land area. Traffic density was equivalent to the total mileage of roads, railways, and waterways in each province divided by the land area. Whether the province has initiated the first level response to the public health emergencies was obtained from the local government website. The results of the descriptive statistics of various variables are detailed in Table 1. The number of confirmed cases in each province is shown in Fig. 2.

Table 1 shows that as of May 2, 2020, the mean number of infected people in various provinces was 2619. In provinces outside of Hubei, the number of infected people was the highest in Guangdong (*n* = 1395) and lowest in Tibet (n = 1). The mean number of people who migrated from Hubei to another province was 318,700; the greatest number of people migrated to Guangdong and the least to Tibet. The mean number of people who migrated from another province to Hubei was 98,910; the greatest number of people was from Henan (*n* = 448,900) and the least was from Ningxia (*n* = 6000). The mean number of permanent residents in each province was 43,950,320 people; the greatest number of people was in Guangdong and the least was in Tibet. The province with the highest population density was Shanghai (3825 people per km^2^), and the one with the lowest population density was Tibet (2 people per km^2^). Shanghai had the highest traffic density, while Tibet had the lowest.

#### Population migration and number of infected people

To determine the relationship between population migration and the number of infected people in each province, the numbers of people who migrated from Hubei to other provinces and vice versa as well as the numbers of permanent residents and infected people in various provinces were tabulated in Table 2. As shown in the second column, the provinces with the highest number of migrants from Hubei, in descending order, were Guangdong, Zhejiang, Shanghai, Jiangsu, Beijing, and Fujian, all of which are more economically developed than Hubei. Meanwhile, the provinces with the least number of migrants from Hubei, in ascending order, were Tibet, Ningxia, Inner Mongolia as well as Liaoning, Jilin, and Heilongjiang (collectively termed the Northeastern China), all of which are the less economically developed regions in the northwest and northeast China. According to the fourth column, the provinces with the greatest number of people who migrated to Hubei were largely from the peripheral provinces of Hubei. They were, in descending order, Henan, Hunan, Guangdong, Chongqing, Anhui, and Jiangxi. The provinces with the least number of people who migrated to Hubei, in ascending order, were Qinghai, Tibet, Ningxia, Jilin, Liaoning, and Tianjin, which are in northwest and northeast China. In contrast, the provinces with the most permanent residents, in descending order, were Guangdong, Shandong, Henan, Sichuan, Jiangsu, and Hebei. Those with the least permanent residents, in ascending order, were Xinjiang, Qinghai, Tibet, Ningxia, and Hainan.

**Table 2:** Hubei-related population migration and numbers of infected people

Rank	Province	PHM	Province	PMH	Province	P	Province	NCOV
1	Hubei	–	Hubei	–	Guangdong	10,724	Hubei	68,127
2	Guangdong	397.34	Henan	44.89	Shandong	9789	Guangdong	1395
3	Zhejiang	124.51	Hunan	28.8	Henan	9436	Henan	1273
4	Shanghai	73.55	Guangdong	20.79	Sichuan	8140	Zhejiang	1218
5	Jiangsu	60.77	Chongqing	20.65	Jiangsu	7960	Hunan	1018
6	Beijing	49.39	Anhui	20.6	Hebei	7384	Anhui	990
7	Fujian	43.96	Jiangxi	17.32	Hunan	6737	Jiangxi	935
8	Hunan	25.08	Sichuan	15.97	Anhui	6083	Shandong	763
9	Tianjin	23.51	Zhejiang	15.59	Hubei	5816	Jiangsu	631
10	Sichuan	15.04	Shandong	13.34	Zhejiang	5508	Chongqing	576
11	Jiangxi	12.49	Fujian	13.06	Guangxi	4754	Heilongjiang	558
12	Yunnan	12.25	Jiangsu	12.33	Yunnan	4714	Sichuan	540
13	Henan	10.93	Hebei	8.63	Jiangxi	4542	Beijing	419
14	Anhui	10.62	Guizhou	8.38	Liaoning	4391	Shanghai	339
15	Shaanxi	10.33	Shaanxi	7.26	Heilongjiang	3833	Hebei	318
16	Shandong	9.76	Guangxi	6.67	Fujian	3806	Fujian	296
17	Hebei	9.4	Yunnan	5.21	Shaanxi	3775	Guangxi	252
18	Shanxi	8.86	Shanxi	4.85	Shanxi	3648	Shaanxi	245
19	Guizhou	8.05	Gansu	4.85	Guizhou	3508	Yunnan	174
20	Guangxi	7.97	Xinjiang	4.38	Chongqing	2991	Hainan	168
21	Xinjiang	7.73	Shanghai	3.37	Jilin	2752	Guizhou	146
22	Chongqing	7.6	Heilongjiang	3.19	Gansu	2591	Tianjin	136
23	Hainan	7.15	Hainan	2.91	Inner Mongolia	2505	Shanxi	133
24	Gansu	4.24	Inner Mongolia	2.74	Shanghai	2426	Liaoning	125
25	Qinghai	3.23	Beijing	2.67	Xinjiang	2298	Jilin	93
26	Liaoning	2.85	Liaoning	2.11	Beijing	2152	Gansu	92
27	Heilongjiang	2.74	Jilin	1.92	Tianjin	1517	Inner Mongolia	77
28	Inner Mongolia	2.73	Tianjin	1.65	Hainan	903	Xinjiang	76
29	Jilin	2.18	Qinghai	1.23	Ningxia	662	Ningxia	75
30	Ningxia	0.99	Tibet	0.76	Qinghai	583	Qinghai	18
31	Tibet	0.85	Ningxia	0.6	Tibet	318	Tibet	1

**Notes:** PHM, number of people who migrated from Hubei to another province; PMH, number of people who migrated from another province to Hubei; P, number of permanent residents in each province; NCOV, number of infected people. “Rank” represents sorting the value of each variable from high to low. The data of PHM and PMH were obtained from the 2015 China 1% National Population Sample Survey, the data of P were obtained from the 2015 China Statistical Yearbook, and the data of NCOV were obtained from the China’s National Health Commission (as of May 2, 2020)

Outside of Hubei, the provinces with the highest number of infected people, in descending order, were Guangdong, Zhejiang, Henan, Hunan, Anhui, and Jiangxi. They could be divided into two categories. The first was the major destinations for population migration from Hubei, including Guangdong, Zhejiang, and other economically developed provinces. The other category was the major origins of population migration to Hubei, including peripheral provinces such as Henan, Hunan, Anhui, and Jiangxi. The least infected provinces were mostly those in northwest and northeast China that were unpopular among Hubei residents, such as Tibet, Qinghai, Xinjiang, Ningxia, Inner Mongolia, and Jilin. This intuitively proved that population migration has a profound effect on the number of infected people in each province, with the major destinations for migration from Hubei and major sources of migration migrating to Hubei as the hardest hit regions. In contrast, northeast and northwest provinces seldom visited by Hubei residents and from which fewer people migrated to Hubei were less affected by the epidemic. To prove the above viewpoint with greater rigor, a quantitative analysis was carried out in the following section. Results are shown in Table 2.

### Data analysis

The empirical strategies of this paper are as follows: on one hand, this study analyzed the impact of population migration factors on the number of infected people. The outcome variable is the number of infected people and the explanatory variables mainly include *P*, *PHM*, *PMH*, *PD*, *TD*, *Over*65 and *Emergency*; on the other hand, this study further analyzed the factors affecting population migration. The outcome variable is the number of population migration, and the explanatory variables include *P*, *neighbor*, *region*, *QHrail*, *dis*tan, *comparison* and *FiveA*.

## Results

### Analysis of migration factors affecting infected people

Contact between the susceptible population and the infected population is the main channel of infection in which population migration plays a critical role. To empirically validate the above deduction, four OLS models were generated (Table 3). In the four models, the dependent variable was the number of infected people. In Model 1, the number of permanent residents in each province was added as an independent variable. In Model 2, the number of people who migrated from Hubei to other provinces and the number of people who migrated from other provinces to Hubei were added as independent variables. In Model 3, the interaction terms added were as follows: (1) between the number of permanent residents in each province and the number of people who migrated from Hubei to that province, and (2) between the number of permanent residents in each province and the number of people who migrated from that province to Hubei. Model 4 was an extension of Model 3, with the addition of variables representing the transmission media, demographic characteristics and response strategies from provinces. Considering that the frequency of contact between the susceptible population and infected population was hardly a measurable condition for human contact, population density and traffic density were used to measure the transmissibility associated with the transmission media. In this study, the focus of the discussion was placed on the regression results in Model 4.

**Table 3:** Population migration-related factors for the number of infected people

Variable	Model 1	Model 2	Model 3	Model 4
P	0.519 (0.462)			
PHM		1.953^***^ (0.452)		
PMH		30.89^***^ (3.438)		
PPHM			0.000115^**^ (0.0000455)	0.0000973^***^ (0.0000335)
PPMH			0.00324^***^ (0.000649)	0.00265^***^ (0.000401)
PD				−0.0992 (0.0632)
TD				202.2^*^ (99.99)
Over65				− 628.6 (1773.9)
Emergency				150.1 (89.33)
_cons	339.2 (829.3)	60.64^*^ (34.48)	194.9^***^ (43.71)	66.53 (129.0)
N	31	30	30	30
R2	0.014	0.831	0.721	0.801

Notes: *, **, and *** represent significance at 10, 5, and 1% respectively; bracketed values denote the standard errors

From Models 1–4, the following findings could be derived: Firstly, Model 1 showed that the permanent population coefficient was positive but insignificant, which suggested that the number of infected people would be zero in provinces without a source of infection. Model 2 showed that the greater the number of people migrating from Hubei to another province or vice versa, the greater the number of infected people in that province. Considering that the outbreak site of COVID-19 was in Hubei Province, the explanation was two-fold in the context of population migration during the Chinese New Year: while permanent Hubei residents from other provinces who were infected returned home during the holiday, infected local residents migrated from Hubei to other provinces for tourism, business, and visiting purposes. This increased the sources of infection in the corresponding provinces. Due to contact between the sources of infection and the susceptible population, every increase of 10,000 in the number of people migrating from Hubei to each province and vice versa would have raised the number of infected people in that province by 1.953 and 30.89, respectively.

According to Model 3, the interaction terms between the number of permanent residents in each province and the number of people who migrated from Hubei to that province, and between the same and the number of people who migrated from that province to Hubei, significantly and positively affected the number of infected people. This validated the theoretical deduction made above that the number of infected people depends on the contact and intensity of contact between the susceptible population and the infected population. In other words, the number of infected people is directly proportional to the product of the number of susceptible people and infectious agents. Considering that the infectious agents are dependent on the transmission media, Model 4 showed that population and traffic density had significant effects on the number of infected people in each province, while the population density, proportion of population over 65 years old and whether to start the first level response before January 24 had insignificant effects on the number of susceptible people in each province. The heavier the traffic density, the more convenient the transportation network, which is conducive to population migration and will accelerate the spread of the epidemic. Therefore, strengthening traffic control plays an important role in controlling the spread of the epidemic.

### Analysis of factors affecting population migration

From the above analysis, it could be concluded that the key factor affecting the number of infected people in each province is the regulation of contact between permanent residents and the infected population in Hubei, that is, population migration is an important factor affecting the number of infected people. Chinese New Year is an important period of population migration in China. During the Chinese New Year, people usually return to their hometowns from their working places for the Chinese New Year (visiting relatives and friends, traveling, etc.), which triggers large-scale population migration in the short term. During the Chinese New Year, many Hubei citizens returned to Hubei from other provinces where they worked. Considering that the outbreak site of COVID-19 was Hubei Province, these uninfected population who mingled with friends and families in Hubei and the probability of these people becoming the source of infection would have greatly increased. Therefore, population migration during the Chinese New Year is an important factor in accelerating the spread of the epidemic. In addition to the special factor of the Spring Festival, there are other factors that have an impact on population migration. Studying the factors affecting the number of people migrating from Hubei to various provinces and vice versa will help to determine the effects of population migration-related factors on the spread, prevention, and control of the epidemic. This is particularly beneficial to the global efforts in pandemic prevention and control, as well as the establishment of an epidemic warning system. Thus, the number of people who migrated from Hubei to each province, the number of people who migrated from each province to Hubei, and the sum of the two were selected as the explanatory variables to generate Models 5–7. OLS regression analysis was then conducted, the results of which are shown in Table 4.

**Table 4:** Factors affecting population migration

Variable	Model 5	Model 6	Model 7
	PHM	PMH	PTH
P	0.00367 (0.00831)	0.000340 (0.000428)	0.00401 (0.00827)
neighbor	−42.48 (35.08)	8.464^***^ (2.735)	−34.02 (33.64)
region	36.42 (22.87)	1.086 (2.184)	37.51 (22.07)
QHrail	1.124 (0.863)	0.138^**^ (0.0582)	1.261 (0.847)
distan	0.0313 (0.0293)	−0.00154 (0.00132)	0.0298 (0.0287)
comparison	45.45^*^ (25.08)	−1.818 (1.929)	43.63^*^ (24.56)
FiveA	31.81 (34.85)	8.588^*^ (4.186)	40.40 (34.77)
_cons	−76.44 (78.76)	3.877 (2.966)	−72.56 (77.56)
*N*	30	30	30
*R*^2^	0.477	0.863	0.526

Notes: *, **, and *** represent significance at 10, 5, and 1% respectively; bracketed values denote the standard errors

From Table 4, the following conclusions could be drawn. First, economic condition is an important factor affecting outward migration from Hubei. A larger number of Hubei residents migrated to more economically developed provinces. Compared with provinces with lower disposable income per capita than Hubei, provinces with higher disposable income per capita attracted, on average, an additional 454,500 people as visitors. Secondly, people from neighboring provinces were more likely to migrate to Hubei. Compared with non-neighboring provinces, they saw, on average, 84,640 more people migrating outwardly to Hubei. A greater number of high-speed trains to and from Hubei was also associated with a greater number of people migrating to Hubei. Every increase of one in the number of high-speed trains would increase the number of people migrating to Hubei by 1380people. People from provinces with more 5A-level scenic spots were also more willing to migrate to Hubei, which may be related to the rich tourism resources in Hubei. Thirdly, the number of migrants related to Hubei Province is only affected by the relative economic level in Hubei Province.

## Discussion

The above theoretical and empirical analyses identified local Hubei residents who migrated to other provinces and residents from other provinces who migrated to Hubei as key enablers of the spread of the epidemic. One of the sources of infection was the population migrating out of Hubei. Economic factors played a crucial role in affecting the outward migration of the Hubei population. Thus, from the perspective of population migration variables, provinces with higher disposable income per capita than Hubei should be regarded as the key regions for epidemic prevention and control, as they were among the top-ranked regions with the most population inflows from Hubei. For example, Guangdong and Zhejiang, both of which had higher disposable income per capita than Hubei, were the first and second most preferred destinations for outward migration by Hubei residents. Large-scale outward migration increases the contact between the susceptible population and infected population, which accelerates the spread of the epidemic. Statistics indicate that, as of 2 May 2020, Guangdong and Zhejiang ranked first and third respectively among provinces outside of Hubei in terms of the number of confirmed cases excluding imported cases. After the outbreak of the epidemic, both provinces were acutely aware of the problem and became the first Chinese provinces to activate the Level-1 Response to Major Public Health Emergencies. They took proactive steps to launch a series of epidemic prevention and control policies, which effectively stalled the spread of the epidemic.

Population migration from other provinces to Hubei constitutes yet another major source of infection. The above theoretical and empirical analyses found that from the perspective of population migration, provinces neighboring Hubei and those that are linked with Hubei via a convenient transport network saw a larger number of people migrating to Hubei. These people were likely to return home during the Chinese New Year, which added to the number of infected people in their home provinces. Hence, epidemic prevention and control efforts should be targeted at these provinces. Henan and Hunan provinces serve as examples. Both provinces are geographically adjacent to Hubei. In terms of transportation, there are frequent high-speed trains operating between both provinces and Hubei, which makes transportation highly convenient. During the Chinese New Year, people migrated from Hubei to the two provinces due to their return home. It is noteworthy that Henan and Hunan were both hit by the epidemic since the onset of the COVID-19 outbreak. As of May 2,2020, the numbers of confirmed cases in Henan and Hunan were the second and fourth highest among Chinese provinces. The Henan and Hunan provincial governments were acutely aware of this problem from the onset, with the former being the more perspicacious. The Department of Public Security of Henan Province immediately established a steering committee for epidemic prevention and control, and the public healthcare sector went into a system-wide state of emergency. At the grassroots level, stringent lines of defense were first set up to minimize the likelihood of any contact between the susceptible population and sources of infection to prevent the rapid spread of the epidemic.

In summary, based on theoretical and empirical analyses of the effects of population migration on the number of confirmed cases in China, in the future China should adopt the following measures to effectively prevent and control the epidemic: Efforts should first be made to specify the directions of outward migration from the domestic origin of infections and define the key regions for epidemic prevention and control based on demographic variables. After locating the key regions, contact between the susceptible population and infected population should be effectively prevented within these regions. The successful outcomes of China’s epidemic prevention and control efforts have attested to the effectiveness of this strategy.

## Conclusion

Our empirical analysis found that population migration and traffic density have a significant positive impact on the number of infected people in China; Further analysis found that the relative economic development level and traffic convenience have an important impact on population migration. China has made remarkable achievements in preventing and controlling the epidemic, which is worthy of reference by other countries. The policy implications of this paper are: there is a need to restrict population migration and reduce human-to-human contact during the epidemic. On the one hand, countries and regions should determine the directions of population migration to and from the epidemic region, and identify whether a region is one of the main destinations or origins of population migration. On the other hand, combined with China’s experience, in terms of city, as Wuhan in Hubei Province was the outbreak site of the epidemic, to prevent the spread of the epidemic caused by population migration, a lockdown was declared, and all transportation links such as airports and railway stations were closed. Public buses, subways, ferries, and long-distance coaches temporarily ceased operations. The lockdown limited the spread of the epidemic to within the city. The shutdown of public transportation in the city minimized human-to-human contact and blocked the spread of the disease within the city.

In addition, the limitations of this study are in the following two aspects: On the one hand, China’s census system is conducted every 10 years but sample survey is conducted every 5 years, and the most recent used in this study were from the 2015 China 1% Population Sample Survey Data. Therefore, the latest data cannot be obtained; on the other hand, this paper is based on Chinese samples, and the conclusions obtained may have limitations and may not be applicable to other countries in the world.

## Data Availability

The datasets generated and analyses during the current study are available in the National Bureau of Statistics of China (http://www.stats.gov.cn/), Chinese Center for Disease Control and Prevention (http://www.chinacdc.cn/) and the official website of the China Railway Corporation (http://www.china-railway.com.cn/), the data was publicly available and the public access to the databases are open.

## Abbreviations

NCOV: Number of infected people
P: Number of permanent residents in each province
PD: Population density
PHM: Number of people who migrated from Hubei to another province
PMH: Number of people who migrated from another province to Hubei
QHrail: Number of high-speed trains between the provincial capital and Wuhan
SEIR: Susceptible-exposed-infectious-removed
TD: Traffic density
WHO: World Health Organization
